# Deploying Viruses against Phytobacteria: Potential Use of Phage Cocktails as a Multifaceted Approach to Combat Resistant Bacterial Plant Pathogens

**DOI:** 10.3390/v14020171

**Published:** 2022-01-18

**Authors:** Tahir Farooq, Muhammad Dilshad Hussain, Muhammad Taimoor Shakeel, Muhammad Tariqjaveed, Muhammad Naveed Aslam, Syed Atif Hasan Naqvi, Rizwa Amjad, Yafei Tang, Xiaoman She, Zifu He

**Affiliations:** 1 Plant Protection Research Institute and Guangdong Provincial Key Laboratory of High Technology for Plant Protection, Guangdong Academy of Agricultural Sciences, Guangzhou 510640, China; tfarooq@gdppri.com (T.F.); tangyf@gdppri.com (Y.T.); 2 State Key Laboratory for Agro-Biotechnology, and Ministry of Agriculture and Rural Affairs, Key Laboratory for Pest Monitoring and Green Management, Department of Plant Pathology, China Agricultural University, Beijing 100193, China; dilshad@cau.edu.cn; 3 Department of Plant Pathology, Faculty of Agriculture & Environment, The Islamia University of Bahawalpur, Bahawalpur 63100, Pakistan; taimoor.shakeel@iub.edu.pk (M.T.S.); naveed.aslam@iub.edu.pk (M.N.A.); 4 Department of Plant Pathology, College of Plant Protection, China Agricultural University, Beijing 100193, China; mtariqjaveed@cau.edu.cn; 5 Department of Plant Pathology, Faculty of Agriculture Science and Technology, Bahauddin Zakariya University, Multan 60800, Pakistan; atifnaqvi@bzu.edu.pk; 6 Department of Bioinformatics and Biotechnology, Government College University, Faisalabad 38000, Pakistan; rizwa.amjad@gmail.com

**Keywords:** plant-bacterial pathogen, anti-phage defense, phage cocktail therapy, polyvalent phage, biocontrol

## Abstract

Plants in nature are under the persistent intimidation of severe microbial diseases, threatening a sustainable food production system. Plant-bacterial pathogens are a major concern in the contemporary era, resulting in reduced plant growth and productivity. Plant antibiotics and chemical-based bactericides have been extensively used to evade plant bacterial diseases. To counteract this pressure, bacteria have evolved an array of resistance mechanisms, including innate and adaptive immune systems. The emergence of resistant bacteria and detrimental consequences of antimicrobial compounds on the environment and human health, accentuates the development of an alternative disease evacuation strategy. The phage cocktail therapy is a multidimensional approach effectively employed for the biocontrol of diverse resistant bacterial infections without affecting the fauna and flora. Phages engage a diverse set of counter defense strategies to undermine wide-ranging anti-phage defense mechanisms of bacterial pathogens. Microbial ecology, evolution, and dynamics of the interactions between phage and plant-bacterial pathogens lead to the engineering of robust phage cocktail therapeutics for the mitigation of devastating phytobacterial diseases. In this review, we highlight the concrete and fundamental determinants in the development and application of phage cocktails and their underlying mechanism, combating resistant plant-bacterial pathogens. Additionally, we provide recent advances in the use of phage cocktail therapy against phytobacteria for the biocontrol of devastating plant diseases.

## 1. Introduction

The immensely expanding human population on planet Earth poses intimidating threats to the food supply chain, which creates ruinous food security risks. To meet the overwhelming demand, a sustainable food production system may need to be developed by reducing the impact of crop diseases. Emerging plant diseases of crops caused by a variety of major phytopathogens, including viruses, bacteria, fungi, nematodes, and oomycetes, are provoking serious challenges, aggravating the global food security system of the contemporary era [1,2,3,4,5,6,7,8,9,10,11]. There are more than 200 species of phytobacteria among phytopathogens that are responsible for significant crop losses during pre-harvesting, storage, and transportation [12]. The most significant are from the genera *Agrobacterium*, *Burkholderia*, *Dickeya*, *Erwinia*, *Ralstonia*, *Pectobacteria*, *Pseudomonas*, *Xanthomonas*, and *Xylella*, which are predominantly evolved to impede plant defense and various control strategies, such as copper-based compounds and antibiotics [13,14,15,16,17,18].

In pathosystem antibiotics, resistance among phytopathogens became a problematic issue when antibiotic application was started at a broader level in the 1940s, after Alexander Flaming’s discovery of penicillin in 1928 [19]. Extensive application may have resulted in the evolution of antibiotic resistance in various plant pathogenic bacteria via horizontal gene transfer-mediated acquisition of resistance determinants. For example, antibiotic resistance genes (*strAB*) are reported to occur in *Pseudomonas syringae*, *Xanthomonas campestris*, and *Erwinia amylovora*, triggering resistance against streptomycin, and these genes are considered to have been acquired from epiphytic bacteria co-located on host plants under antibiotic selection [18,20,21,22,23].

The widespread use of copper-based antimicrobial pesticides may result in its accumulation in the environment and food crops, which has been associated with human health hazards, toxic effects on plants, and the evolution of copper-tolerant phytopathogens [24,25,26,27,28,29]. Copper-induced toxicity has primarily been associated with several human and animal concerns, including reproductive, hepatic, gastrointestinal, and neurodegenerative disorders [30,31,32,33]. Furthermore, copper-mediated intoxication has also been reported to increase the mortality and morbidity of *Drosophila melanogaster* and *Apis mellifera* [34,35,36]. An excessive application of copper pesticides and copper-oxide nanoparticles on various agricultural crops, such as *Brassica chinensis*, *B. alboglabra*, *Chrysanthemum coronarium*, and *Hordeum sativum* distichum, may result in oxidative stress, impairment of growth, photosynthetic pigment deterioration, and germination cessation [37,38,39,40]. However, phytopathogens have evolved to develop resistance against the aforementioned widespread application of pesticides, which has become a serious challenge of the current scenario in the agricultural production system [27,41]. Plant pathogenic bacteria included in the genera *Stenotrophomonas*, *Xanthomonas*, and *Pseudomonas*, are resistant to copper-based antimicrobial pesticides, threatening microbial control strategies [42,43,44,45]. Similarly, *X. citri* subsp. *citri*, *X. alfalfae* subsp. *citrumelonis*, *X. euvesicatoria*, *X. perforans*, and *P. syringae* pv. *phaseolicola* have exhibited copper resistance and have caused severe diseases in citrus and tomato crops [42,46]. As copper application is regarded as a primary approach to control phytopathogens, it is a huge concern in microbial disease management. Recently, copper-based plant protection compounds have been banned or limited in several countries, and innovative control strategies, including the recruitment of bacteriophages as potential sustainable antimicrobial agents, have been established [47,48,49,50,51,52].

**Figure 1:**
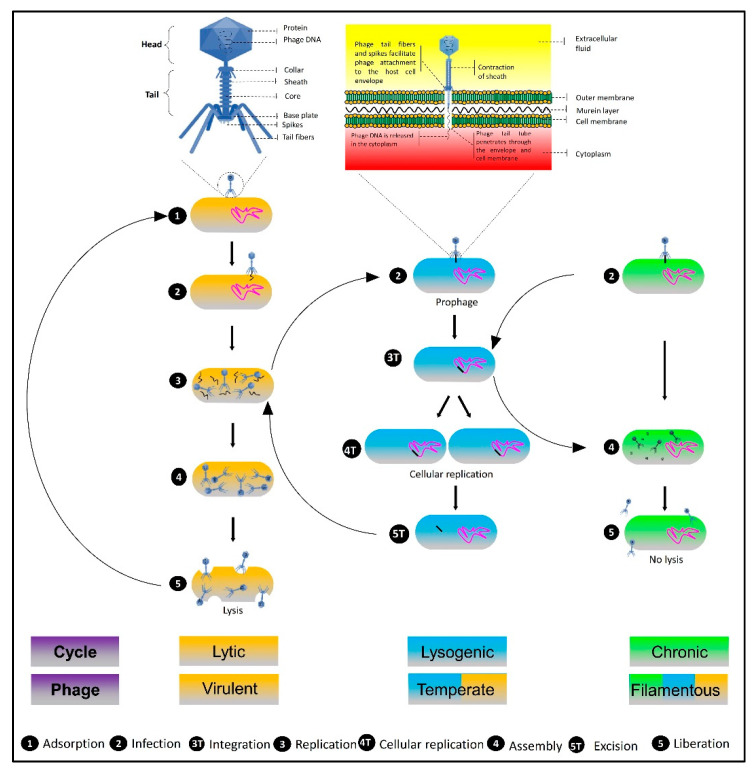
Schematic illustration representing the bacteriophage particle morphology, mechanism of penetration in the host bacterial cell, and different types of life cycles (created with BioRender.com, accessed on 23 November 2021).

Bacteriophages (phages) are viruses specifically infecting and replicating in bacteria as antimicrobial agents, leading to the degradation of bacterial hosts. Phage therapy is a promising multifaceted approach to combat resistant bacterial plant pathogens for the management of bacterial disease to improve crop productions [52]. Frederick Twort and Felix d’Herelle independently discovered bacteriophages in 1915 and 1917, respectively, after 20 years of virus discovery. The antimicrobial characteristics of phages were immediately recognized by Felix d’Herelle in 1919, demonstrating his phage preparation aptitude to treat dysentery patients in the Hôpital des Enfants-Malades in Paris [12,53,54]. The benign nature of phages to eukaryotic cells, host specificity, self-replication, capability to overcome resistance, and ease of biosynthesis are all factors that have sparked interest in them as biocontrol agents [52]. Their omnipresence and abundance in the biosphere enable their isolation from their surroundings. Phages are tadpole-shaped; a polyhedral head, a short neck with collar, and a straight tail are the hallmarks of their morphology. A bipyramidal hexagonal-shaped head encloses highly folded double-stranded DNA with a capsid assembled by 2000 capsomeres (proteins), and a hollow cylindrical tail consists of a central core enfolded by a contractile sheath. The phage genome enters the host cell through the central space of the core [55,56] (Figure 1). The order *Caudovirales* is comprised of three-tailed phage families, such as *Podoviridae*, which have short non-contractile tails, *Siphoviridae*, which contain long flexible tails, and *Myoviridae*, which have rigid contractile tails [12].

Phages can replicate via lytic (virulent), lysogenic (temperate), and chronic (filamentous) life cycles, which are their most important determinants and confer incredible biocontrol characteristics. A phage infects a bacterial host via interacting with receptors on the cell, and then adsorbing and injecting its DNA into the host cell. The subsequent strategy of phage replication depends exclusively on whether it is virulent or temperate. Virulent phages (phage T4) infect and use the host cell metabolism to replicate via the lytic cycle, a process involving the lysis of host cells and the release of new phage progeny. Alternatively, temperate phages, such as phage λ, infect the host cell, enter either the lytic cycle, resulting in episomes, or integrate into the bacterial genome, termed prophage, in the process called lysogeny or the lysogenic cycle [57]. Lysogeny triggers the replication of prophage in association with the host genome either in an isolated plasmid-like state (phage P1) or incorporated into the bacterial chromosomes (phage λ). These prophages can exit the lysogenic cycle under unfavorable conditions and produce ample virions through the lytic cycle-mediated cell lysis [54,58]. Filamentous phages (phage M13) may reside in the temperate or virulent phages of the host cell and proliferate via the chronic cycle under stress conditions. These phages are either secreted from the host without cell lysis or transferred horizontally with cellular division [59] (Figure 1).

Recently, increased copper and antibiotic resistance, along with a scarcity of novel antimicrobial medicines, has sparked a revival of phage-inspired antibacterial strategies, termed as phage therapy, in agriculture, medicine, and several food industries. Phage therapy via employing natural or engineered virulent phages, such as phage cocktails, has offered a highly effective biocontrol of wide-ranging plant bacterial diseases [12,49,60,61]. Consequently, phage cocktails are novel and potentially sustainable antibacterial genetic entities to combat various resistant bacterial pathogens, including *Xylella* *fastidiosa* subsp. *fastidiosa*, *Ralstonia* *solanacearum*, *P. aeruginosa*, and *E. amylovora* [61,62,63,64]. However, their engineering and intricate antimicrobial interactions are a matter of consideration. Phage therapy is a game-changer technology in agriculture, food industries, and clinical therapeutics, but it needs considerable concentration from the scientific world to develop eco-friendly biological control strategies for microbial pathogens. This communication explores new insights into the formulation of effective phage cocktails and factors that influence their development and applications. Additionally, it highlights the underlying mechanism of interactions between phages and resistant bacterial pathogens, facilitating the engineering of efficient phage cocktail therapeutics against phytobacteria for the biocontrol of overwhelming plant diseases.

## 2. Phage Cocktails as Antibacterial Therapeutic Agents

Phage cocktails are ubiquitous attractive antimicrobial agents with exceptional major characteristics of specificity and exponential proliferation. A cocktail of phages that demonstrate wide-ranging host activity, reflects a diversity of receptors that might exploit the potency of the antibacterial therapeutics and curtail the possibilities of resistance development [65,66]. Biologically engineered phage cocktails can be used as a natural biocontrol for several bacterial diseases, targeting resistant pathogenic bacteria without harming the host plant or animal and their commensal microflora. Although phage-based biocontrol approaches still have to be explored, it is primarily accepted that temperate phages should not be considered for therapeutic application due to their propensity for specialized or generalized pathogenesis-determinant transduction [67,68]. Phage cocktails have great promises in biocontrol of resistant bacterial infections of plants and animals. For example, gamma-proteobacterium, *X. fastidiosa* subsp. *fastidiosa*, poses severe intimidation to the wine industry in the United States of America by causing Pierce’s disease (PD) of grapevines. An engineered cocktail of four lytic phages is reported as an effective therapeutic agent for the biocontrol of *X. fastidiosa* and its associated infections, including PD, olive-quick decline syndrome, and oleander, almond, or coffee leaf scorch [62,69,70]. Similarly, a cocktail of phages is widely used to control antibiotic-resistant *Staphylococcus aureus* and *P. aeruginosa* in the treatment of chronic otitis infections [64]. In the food industry, phage cocktails are used as an innovative therapeutic approach to treat various foodborne pathogens, such as *Salmonella entrica*, *Listeria monocytogenes*, *Escherichia coli*, *Shigellasonnei*, and *Campylobacter jejuni* for pre- and post-harvesting food protection and processing to save millions of lives from food poison-mediated diseases [71,72,73,74]. P1 cocktail consisting of six phage isolations may kill 98% of *R. solanacearum* through direct application via soil drenching for the biocontrol of bacterial wilt disease of potato and tomato [61,75]. In addition, phage cocktails can be applied in a variety of ways, such as foliar spraying, soil drenching, infiltration, and immersion. Consequently, phage cocktails have been reported as effective biocontrol agents to reduce the incidence of several diseases, including bacterial blight in leek caused by *P. syringae* pv. *porri* [76], black rot of broccoli caused by *X. campestris* pv. *campestris* [77], bacterial spot of pepper caused by *X. euvesicatoria* [78], and bacterial soft rot of onion caused by *Pectobacterium carotovorum* subsp. *carotovorum* [79]. The applications of phage cocktails as antimicrobial agents have revolutionized biocontrol strategies in integrated disease management of resistant microbial pathogens without wreaking havoc on fauna and flora. However, concrete efforts are required to enhance their optimal engineering and control efficacy of resistant phytopathogens, and facilitate their availability at a broad spectrum for field applications.

## 3. Methods and Considerations for the Development of Effective Bacteriophage Cocktails

**Figure 2:**
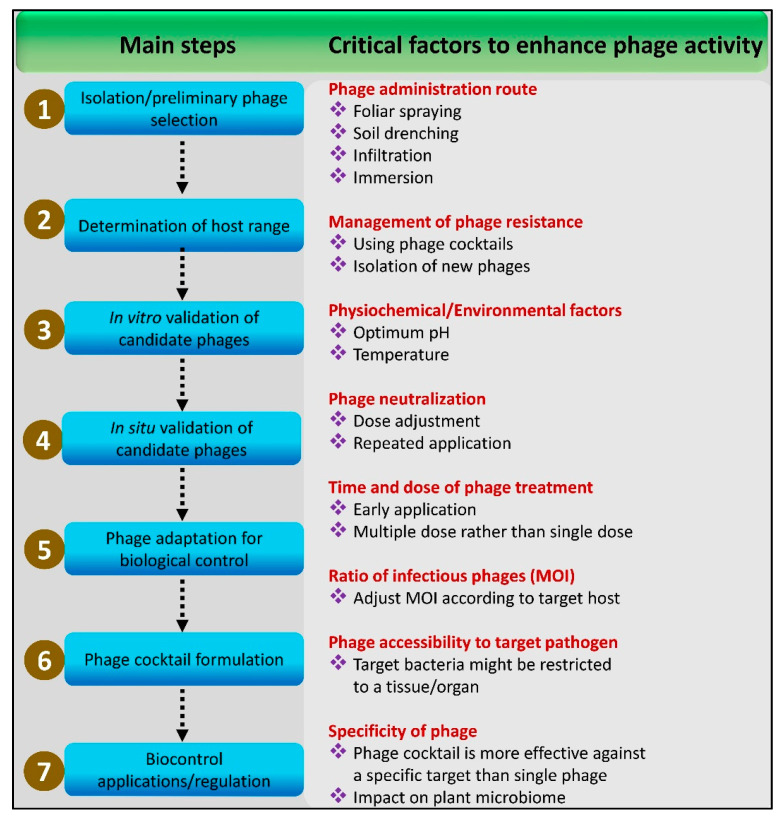
Steps and critical factors involved in the isolation, characterization and optimization of bacteriophages to achieve maximum biocontrol efficacy against plant pathogenic bacteria.

As mentioned above, phages habitually demonstrate host specificity. This nature may limit the application of a sole phage in the field when targeting different plant pathogens at the same locality. Therefore, a developed cocktail of phages is mandatory for the biocontrol of diverse resistant phytobacteria under field conditions. A cocktail of lytic phages isolated from various sources could have the potential to be exploited as a universal antibacterial biocontrol agent with minimal risk of resistance development. Idyllically, phages in a cocktail would cover the broadest possible spectrum of target pathogens and have a distinct mechanism of infection while ensuring complementary pathogenic potential on the host–pathogen interface [52,54,75,80,81]. Phages with diverse receptors, strong adsorption, short latency, and huge burst size ought to be considered during phage cocktail formulations. Polyvalent phages, with a broad host range that can infect multiple bacterial strains belonging to the same species, may also be accounted for in the formulation of phage cocktails [82,83]. In the formulation of phage cocktails and their biocontrol applications, there are several developmental steps involved, such as isolation and characterization of phages with broad host-range, in vitro and in situ validation of candidate phages, and phage adaptation for biocontrol therapeutics (Figure 2). Regardless of the design used to develop phage cocktails, it is mandatory to assess a phage’s in vivo or in vitro therapeutic potential, including its host range activity, genomic features, adaptations for biocontrol, storage and application requirements, and efficiency against pathogens [84,85,86,87,88]. There are various approaches addressing all of these developmental steps for the formulation of effective phage cocktail therapeutics. Several polyvalent phages are isolated from different environmental samples, which are in direct contact with the targeted host bacteria. These may be sewage samples, water samples, raw fecal matter, soil samples, infected plants, and clinical samples, from which, polyvalence phages are isolated for the development of effective bacteriophage cocktails employing various multi-dimensional approaches [61,75,83,89,90,91,92,93,94].

### 3.1. Step-by-Step Method

SBS is the most significant and generally accepted approach for the development of phage cocktails with inordinate therapeutic potential to combat multidrug-resistant bacterial pathogens. In this method, wild-type lytic bacterial strains and wild-type phage resistant mutants are used to isolate the phage employed in the development of a phage cocktail. The wild-type bacterium is rendered insensitive to the first phage, resulting in a phage-resistant mutant. Thereafter, this phage-resistant mutant is utilized further to isolate the second phage. Similarly, the third phage may also be isolated by using bacterial mutant resistance to the second phage. Finally, all of the phages are combined into a cocktail that can inhibit the development of phage-resistant bacteria [95]. For example, a tri-phage cocktail (GH-K1, GH-K2, and GH-K3) established by the SBS method has great therapeutic efficacy against mono-phage-resistant *Klebsiella pneumoniae* and reduces its resistance-triggering mutation frequency [90]. This method may be exploited to locate rich sources of phage, which can be engineered to overcome the phage resistance in phytobacteria. However, this approach can be laborious, notably when targeting diverse phytopathogens. Therefore, to achieve effective biocontrol, it would be indispensable to target all the distinct phytopathogens.

### 3.2. Targeting Phage Receptors

The identification of phage receptors in a pathogen is the most imperative strategy in the selection of phages involved in the development of a cocktail. Phage receptors mediating the adsorption of phage to host cells determine the susceptibility of bacteria to phage infection. These receptors include outer membrane proteins, lipopolysaccharides (LPS), pili, flagella, capsules or slime layers, and wall teichoic acid (WTA) [96,97,98]. For example, *R. solanacearum* threatens several crops globally. However, mutations in *R. solanacearum* GMI1000 loci (*RSc2958-RSc2962/mla*) that trigger LPS biogenesis may regulate phospholipid trafficking in the outer membrane and peptidoglycan recycling can protect the mutants from the adsorption of phages on the O-antigen [97]. In previous studies, it was reported that phage receptors in *Yersinia pestis* were found across the LPS core. Knockout bacterial mutants were generated by site-directed mutagenesis of genes involved in the production of different parts of LPS. Thereafter, these genes regulating LPS biosynthesis were cloned into vectors and utilized in trans-complementation tests to determine their susceptibility to various phages [95,99]. This dynamic approach can be used to develop phage cocktails for the biocontrol of different phytopathogenic bacteria. Moreover, for the development of phage cocktails, the functions of exoploysaccharides (EPS) in phage–host interaction must be considered, because composition and expression level of EPS determines the pathogenesis of virulent phages [100,101]. Phages from the *Myoviridae* and *Podoviridae* families are lytic for the *E. amylovora*, but their infectivity is associated with EPS level and composition. For example, *Myoviridae* phages preferentially infect low or acidic EPS (amylovoran), producing hosts, while *Podoviridae* phages demonstrate a preference of infectivity to the hosts, producing high or neutral EPS (levan) [101]. In the formulation of phage cocktails, it is mandatory to target conserved receptors involved in the survival and infectivity of plant pathogenic bacteria.

### 3.3. Phage Lytic Curve Approach

A phage lytic curve approach can also be used to assemble phage cocktails by selecting phages from a phage agglomeration based on phage lytic or lysis curves. A phage lytic curve is a measure of lytic phages’ antibacterial activities [75,102,103]. The lytic curves are generated by continually computing the optical density of pathogenic bacteria in their exponential phase infected with phage(s) at a certain concentration for a predetermined period [95]. The phage(s) that lead to reduced bacterial optical density, demonstrating different lytic curves, are selected for the formulation of phages with vigorous lytic activity. Another phage score method is also established to describe phage lytic activity against the bacterial host. In this method, two model organisms, such as gram-negative *E. coli* and gram-positive *Staphylococcus aureus,* are cultivated under controlled conditions with three T_4_-like wild-type phages for *E. coli* and three lytic phages infecting *S. aureus* in association with different initial multiplicity of infection (MOI, ranging from 0.01–1 for *E. coli* and 0.1–1 for *S. aureus*) [104]. Through employing mathematical expressions, the phage score method provides significant tools for the characterization and comparative evaluation of the lytic activity of phages.

### 3.4. Application of Host-Range Mutant Phages

Host-range mutant (H-mutant) phages are vigorously used in the formulation of phage cocktails with an expanded host range. Moreover, H-mutant phages may also be involved in the characterization and determination of the host range [76,105,106,107,108,109]. Experimentally, bacteria are plated on double-layer agar with a broad host range phage and incubated to produce h-mutant phages. This method finds a phage-resistant mutant, which is then exposed to a high phage concentration, plated on double-layer agar, and incubated. The appearance of plaques on the double-layer agar leads to the selection of a mutant phage capable of lysing both wild-type and phage-resistant mutant pathogenic bacteria [95,106].

### 3.5. CRISPR-Cas System

Genetically engineered phages may also be used to develop synthetic cocktails to target certain plant bacterial species within a mixed population [110,111]. There are several technologies, such as CRISPR-Cas-mediated genome engineering, homologous recombination, bacteriophage recombineering of electroporated DNA (BRED), in vivo recombineering, rebuilding/refactoring phage genome in vitro, yeast-based assembly of phage genome platform, cell-free transcription–translation system, and whole-genome synthesis from synthetic oligonucleotides, involved in the genetic modification of phages [112,113]. For example, through homologous recombination, *E. amylovora* phage Y2 can be engineered by introducing bacterial luxAB fusion or *E. amylovora* phage L1 depolymerase gene into the genome of phage Y2, which leads to increasing its killing efficiency. The genetic modification of phage receptor binding proteins may also enhance the host range [95]. Similarly, the CRISPR-Cas system is frequently used in the engineering of phages. It was used to edit the genome of T7 phage for the first time in 2014 [114]. Recently, CRISPR-Cas of *Listeria monocytogenes* is being used as an emerging platform for the engineering of *Listeria* phages [112,115]. Thus, these synthetic phages are also involved in enhancing the lytic activity and determining the host range of various phages.

### 3.6. Phage–Bacteria Infection Networks

Recently, phage–bacteria infection networks (PBINs) have been developed by two algorithms, such as genetic (nestedness temperature calculator) and heuristic (BinMatNest), which is a newly emerging pipeline to design phages. It simplifies phage identification of the broadest host range [116]. Host range matrices have never been transformed into PBINs for designing phage cocktails. This novel approach optimizes the cocktail formulation and reduces the complexity of phages, because excessive phages may lead to horizontal genes that expand the fitness of host strains, dysbiosis, and manufacturing costs.

### 3.7. High-Throughput Sequential Platforms

The antibacterial resistance dilemma has rekindled interest in phage therapy as an alternate method of infection treatment. However, traditional approaches for isolating phages are time-consuming, laborious, and usually dysfunctional. Recently a computational approach has been established to meet the overwhelming demands of therapeutic cocktails [117]. The computational bases include Islander [118] and TIGER [119], which locate and map integrative genetic elements (IGEs) within bacterial and archaeal genome sequences, leading to demonstrate bacterial hosts, complete sequence, and each prophage end sequence. Thus, prophage sequences enable its genome engineering to develop safe therapeutic cocktails combating the unscrupulous pathogen *P. aeruginosa* PAO1 [117]. This technological platform may provide new insights in synthetic biology for the rapid development of therapeutic cocktails against several pathogens, if not all plant pathogenic bacteria, paving the way for the application of phage therapy against infectious diseases. In the case of plant bacterial treatment, it needs further attention to develop a precise biocontrol cocktail therapy against threatening bacterial diseases.

However, the above-mentioned phage cocktail formulations are not feasible to thoroughly avoid the emergence of new phage-resistant phytopathogens due to the never-ending arms race between phages and bacterial pathogens. It is also worth emphasizing that due to the complexity of plant–pathogen systems, a single multidimensional cocktail for all bacterial phytopathogens may not be plausible to develop. Therefore, it needs constant surveillance of phytopathogens and modification of phage cocktail formulations to ensure the target of newly emerging phage-resistant plant pathogenic bacteria.

## 4. Critical Factors in the Development and Application of Phage Cocktails

The characteristics of cocktail therapeutics are associated with the phages used in therapy. These include phage virion stability, phage receptor secretion, impeding bacterial virulence factors, and host pathogenicity, all of which are in favor of phage cocktail therapy. However, there is an encoding of bacterial virulence determinants and a propensity to produce bacterial lysogens, both of which are detrimental to phage therapy [67,81,84,120]. Furthermore, there are concerns about whether antibacterial therapy applied as mono-phages rather than multi-phages cocktails will interact with each other’s ability to generate new virions when infecting the same bacterium, whether bacterial resistance to individual phages will or will not eagerly evolve in vivo or in vitro, and whether bacterial mutations to phage resistance may lead to suppressing bacterial fitness or virulence [121,122,123,124,125,126,127,128]. Therefore, several factors influence the formulation and application of phage cocktails.

### 4.1. Long-Term Storage and Transportation

The antimicrobial effectiveness of phage cocktail therapeutics may be negated by several factors influencing their course of development and applications. Several key factors are considered to significantly undermine their biocontrol efficacy [129,130,131,132,133,134]. Phages may gradually lose their activity when they are stored for a long duration under ambient conditions, necessitating the use of stabilized formulations and the conversion of aqueous phage formulations to powder form. Primarily, phages are composed of genetic material encased by a protein capsid (protein-rich coat of phages, as well as their complexity, makes them vulnerable to external storage conditions), which interacts with each other via intermolecular interactions. Therefore, high-level conversion of aqueous phage formulation into powder phase for long-term storage or transportation may be prohibited because it adversely affects their viability. The stabilizers, including polyethylene glycol (PEG) and sucrose in lyophilized phage, have not been confirmed to be effective for long-term storage [135]. In addition, none of the stabilizing approaches or formulations explored so far appear to be universal, due to the variable susceptibility of particular phages to chemical and physical factors, such as acidity, ions, and temperature. The stability of phages is correlated with external factors. Most phages can be stored in an aqueous or lyophilized form for an extended time at neutral pH levels ranging from 6 to 8 [136]. In general, phage titers are decreased gradually with pH. For example, the phage titer of *S. aureus* is reported to decrease by 2 logs within 4 to 6 hours as the pH changed from 6.19 to 5.38. A pH lower than 4.5 may impede the proliferation of several phages. For example, the phage PM2 from the *Corticoviridae* family completely loses its viability after 1 hour at pH 5.0 at 37 °C. Phage T4 from the *Myoviridae* family is not stable at pH < 5. Phages of *Lactococcus* can survive at high temperatures (40–90 °C). Furthermore, phages can be stored for a long duration when stored at refrigerator temperature [137,138]. It is reported that NaNO_3_ electrolyte does not influence the stability and titer of the F-specific RNA phages (MS2), and the ionic strength may enhance the aggregation of phages [139,140]. Thus, in a cocktail, each phage may have variable storage conditions, and it has become an emerging challenge in synthetic biology for the long-term storage of phage cocktails developed by phages of varying sensitivity. For the effectiveness of phage cocktail therapeutics, it is mandatory to maintain the viability of individual phages over a specified duration of storage.

### 4.2. Adverse Environmental Conditions

In the phage cocktail-based biocontrol of plant diseases, the transient persistence of phages in diverse plant environments is still a major challenge [141,142,143,144,145]. Probably, most practicing approaches of phage cocktail application to plant systems are spraying, drench, or drip application [12,141,144]. These methods expose phage cocktail biocontrol agents to adverse rhizosphere and phyllosphere environmental conditions. In the rhizosphere, heterogeneous soil matrix, moisture, and soil pH inhibit phage diffusion in soil and may prevent the use of phage cocktails as biocontrol agents. In the phyllosphere, phages are exposed to sunlight and unfavorable temperatures, which adversely reduce the effectiveness of phage cocktails. Therefore, the phyllosphere is more destructive to phages as compared to the rhizosphere. To achieve the efficacy of these biocontrol agents in the phyllosphere, several techniques have been investigated to increase phage viability and survival, such as protective formulation, avirulent bacterial carriers, and phage cocktail application in the early mornings and evenings [144,146,147]. For example, the *X. perforans* attenuated strains increase the phage cocktail persistent on the leaf surface of *Solanum lycopersicum* and sustain the population of phages without affecting the plant development and phage cocktail viability [106,107,144]. Several protective materials, such as pregelatinized corn flour (PCF), skim milk, and casecrete in specified formulations protect the phage from adverse environmental conditions and improve their effectiveness [146,148,149,150,151,152]. For instance, natural compounds in a formulation of 5% red pepper juice or 10% carrot, 34% and 28%, respectively, of the initial phage titer protect the *E. amylovora* phage Y2 from 5-min exposure to UV irradiation. Furthermore, 50 mM phenylalanine, 50 mM tryptophan, and 50 mM tyrosine in a ratio of 1:1:1 may also increase the viability of the phage [148]. These formulations are very critical in phage cocktail therapy, which makes it laborious and time-consuming. The use of avirulent bacterial strains with phage in equivalent numbers may be a challenge in wide-host range cocktails. Therefore, precise strategies are needed to promote the biocontrol mechanism of plant pathogenic diseases.

In phage therapy, the escape of invading pathogens into confined tissue and organ compartments may obstruct the successful use of phages, particularly if the phage cocktail cannot aggressively chase the bacterium. Therefore, it is questionable how efficient phages would be in addressing disease induced by intracellular pathogens including *Salmonella* species [153,154]. Mostly, phage cocktails cannot diffuse across the membrane-like antibiotic molecules and thus require a mechanism of administration to reach a specific target cell. To attain an efficient mechanism of delivery, non-pathogenic bacteria may be used as a vehicle to transport the phage to its pathogenic target [131].

### 4.3. Affecting Plant Microbiome

Complex phage cocktails, containing polyvalent phages, may pose a serious threat to non-targeted bacterial communities (plant microbiome), even if the impact is minimal. Polyvalent phage cocktails are applied as antimicrobial agents to target several plant pathogenic bacteria and may end up killing endophytes. Polyvalent phages are expected to influence the composition of a plant microbiome either directly by affecting the evolution or population size of a microbial community or indirectly by shaping competition among species within the plant [12,155]. Moreover, the plant microbiome may be hostile to the action of a phage cocktail if it is antagonistic to phytopathogens. Consequently, the plausible influence of phage cocktails on the plant microbiome during field trial investigations may be explored by employing quantitative molecular approaches before and after phage cocktail application.

### 4.4. Time and Cost of Development

The time and expense of developing, evaluating, and modifying relatively complex phage cocktails are key factors affecting the development and application of phage cocktails. Although phages may be isolated in a matter of days, comprehensive characterization, purification, validation, and formulation of large numbers of phage cocktails can be time-consuming and expensive [110]. The cost, however, would be determined by the number of phytopathogens addressed. Phage banks can be used to hold freshly isolated and described phages, reducing the time required to design and formulate phage cocktails. Plant protection products or biopesticides must meet regulatory standards, such as safety, reliability of efficacy, and quality. Legislative standards vary from country to country and may necessitate new approvals and certification when a phage cocktail is modified, as it may be deemed a new product [95,156]. Although scientific standards must be maintained, there is a need for regulatory framework versatility to allow for the swift updating of phage cocktails in response to the emergence of phage-resistant phytopathogens.

## 5. Types of Phages Used as Biocontrol Agents and Underlying Mechanisms of Action

From a historical perspective, the term phage therapy was premeditated for animal and human therapeutics, but nowadays phage biocontrol strategy is more frequently used against a large number of devastating plant bacterial pathogens with significantly promising consequences [12,62,76,82,157,158,159,160,161]. The main consideration in determining whether a phage is suitable for biocontrol is whether it is predominantly lytic or temperate in nature. Virulent phages induce the infection that eventually ends with the lysis of the host bacterium, releasing progeny phage particles. Temperate phage can invade the host via lytic route, but can also infect through the lysogenic pathway, in which the genome of phage integrates into the chromosomes of the host bacterium or persist as a prophage [162,163]. However, filamentous phage genome may proliferate exclusively within the host or following the lysogenic route of infection and phages consistently extruded across bacterial membranes without being lysed.

### 5.1. Filamentous Phages

Filamentous phages are single-stranded DNA (ssDNA) viruses from the family *Inoviridae*, which can infect several gram-negative bacteria included in the genera *Salmonella*, *Xanthomonas*, *Escherichia*, *Pseudomonas*, *Vibrio*, *Neisseria*, and *Thermus* [59,164,165,166]. Filamentous phages are also employed as biocontrol agents against plant bacterial infections. In one study, it was reported that the filamentous phage XacF1 can invade *X. axonopodis* pv. *citri* at *dif* site (*att*B) through the host XerC/D recombination. Fascinatingly, the infection with XacF1 results in various physiological alterations in host cells, such as the downregulation of EPS production, restricted motility, delayed growth rate, and a significant decline in pathogenicity. Particularly, the reduction in pathogenicity demonstrated that XacF1 may be used as a biocontrol agent against citrus canker disease [167]. The underlying mechanism of infection requires the binding of filamentous phage to its host. Phage (M13, fd, f1) adsorption is triggered by the binding of a phage-encoded protein (gene 3 protein, g3p) to the host cell surface receptor, namely, the F-pilus of *E. coli* cells. Subsequently, g3p binds to ToIA, an inner membrane protein that serves as a co-receptor. Phage genomic ssDNA is translocated into the host cytoplasm, stripping itself of the major-coat protein (gp8). The complementary DNA strand of the phage genome is produced in the host and then incorporated into the host’s genome by exploiting the host’s machinery. In such cases, the phage genome remains inactive until stress signals trigger replication to synthesize circular, supercoiled double-stranded DNA (dsDNA), also named as the replicative form (RF), with the integrated genome serving as a template. Some filamentous phages (M13) have genomes as plasmid-like vectors, and their DNA is immediately transformed to RF by host-encoded enzymes. The phage’s genome may be transferred horizontally with the cell division, similarly to other plasmids, or by recombination [166,167,168,169]. Filamentous phages may reside in the host cell as either temperate or virulent phages, and integrate themselves into the host chromosomes as prophages, playing significant roles in the virulence and evolution of pathogenic bacteria as reported with the plant pathogen *R. solanacearum* with its phage ϕRSS1, which causes increased virulence. Therefore, their precise role as biocontrol agents is still a matter of consideration.

### 5.2. Temperate Phages

As above-mentioned, temperate phages can enter the lysogenic route of infection in which their DNA genomes integrate into host chromosomes as prophages and multiplicate in synchrony with host chromosomes [170,171,172,173,174,175,176]. Temperate phages can enter into the lytic cycle by spontaneously switching from lysogeny under physical or chemical environmental cues (UV-light or heat). Recently, Al-Anany et al. reported that the temperate phage HK97 and antibiotic ciprofloxacin combined application results in the eradication of bacterial pathogens [177]. This phage–antibiotic synergy (PAS) increases the effectiveness of the phage against several bacterial pathogens [178,179,180,181,182]. Furthermore, it is a diverse mechanism that does not simply enhance the phage production and operates through the RecA protein, but is also a critical component of the bacterial SOS response [177]. This strategy may somewhat strengthen their candidature for biocontrol agents but it needs serious attention in this regard [183]. On the other hand, prophages are considered as evolutionary molecular time bombs, because prophage DNA can play a significant role in the evolution and emergence of novel pathogenic strains via transduction or horizontal transmission of virulence genes among bacteria [57,184,185,186,187,188]. For example, in the case of phytopathogens, prophages of the *P. atrospeticum*, such as ECA29 and ECA41, both increase the bacterial host motility [189]. Therefore, suitability of both the filamentous and temperate phages for biocontrol applications is questionable, as their infection can have varied impacts on host virulence.

### 5.3. Lytic Phages

Ideally, a candidate phage for biocontrol therapeutics should be exclusively lytic with a wide-host ranging capability that enables an efficient infection on all resistant pathogenic bacterial strains, including the genus and species being targeted. Furthermore, the prevailing thought is that phages should be capable to lyse the host swiftly while creating a large number of phage progeny and diffuse efficiently through the environment in which they are being administered. Phages included in the *Caudovirales* infect the host through an immediate expression of discrete genes followed by hijacking the host cellular machinery and diverting it to phage DNA replication and protein synthesis, triggering the pathogenicity of these phages that degrade the peptidoglycan (PG), leading to host cell lysis, death, and progeny release for succeeding infections [190]. The pathogenicity of three lytic phages, including vRsoP-WF2, vRsoP-WR2, and vRsoP-WM2, was confirmed in a variety of realistic situations and host ranges, inducing a successful infection in the targeted plant pathogens, such as *R. syzygii* subsp. *indonesiensis*, *R. solanacearum*, or *R. pseudosolanacearum,* and exhibiting features suited for biocontrol application [12,47]. Similarly, *Podoviridae*-like lytic phages, such as RsoP1IND, ϕRSA1, ϕRSB1, and ϕRSL1, are also effectively employed in the biocontrol of bacterial wilt caused by *R. solanacearum*, although their infectivity is associated with the EPS level and composition. Consequently, *Podoviridae* phages preferentially infect hosts that produce high or neutral EPS [47,101,191,192,193,194]. Lytic phages interact with the host through completely different mechanisms than antibiotics, having extraordinary effectiveness against both antimicrobial resistant (AMR) and non-AMR bacterial infections [195]. For example, lytic phages can interact with SAR inducers to integrate into the host for successful management of tomato bacterial spot and Xanthomonas leaf blight of onion [50,52]. Lytic phages have prime importance in agriphage cocktail formulations for sustainable disease management to enhance crop production. Considerable attention is required to study their underlying mechanism of infection due to the diversity and interaction of each phage in a cocktail.

## 6. Recent Advances in the Use of Phage Cocktail Therapies against Phytobacteria

**Figure 3:**
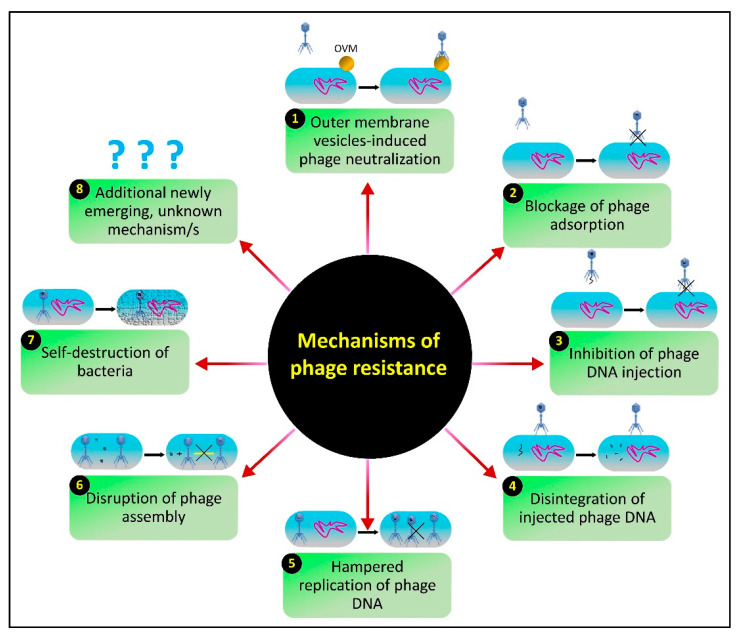
A schematic representation of multiple pathways by which phytopathogenic bacteria can achieve phage resistance.

Phage cocktail therapy is a biotechnologically designed biocontrol multidimensional approach for sustainable disease management, capable of targeting resistant phytobacteria with extremely high efficiency [12,52,95,196,197,198]. As mentioned earlier, the co-evolution of phages and their hosts has brought several phage-resistant mechanisms (Figure 3), which make phytobacteria invulnerable to phage therapy. Indeed, it is a big challenge in sustainable disease management, which needs to addressed. *E. amylovora*, for instance, is a devastating pathogen of various destructive bacterial diseases of the family *Rosaceae* and several economically important fruit trees, such as pear and apple trees. To avoid the infection, *E. amylovora* produces EPCs as a physical barrier that subverts cell surface receptors, inhibiting phage adsorption and rendering the bacterium immune to phage infection [8,199]. Recently, a SBS method has enabled phage cocktail formulations, including a combination of three phages, ϕEa2345-6, ϕEa1337-26, and Eh21-5, from *Myoviridae* and *Podoviridae,* against fire blight of apple and pear, and a cocktail of four *Myoviridae* phages, Eram2, Eram26, Eram24, and Eram45, against fire blight of pear. These engineered phage cocktails are being applied vigorously to achieve effective control of *E. amylovora* [198]. These phage cocktails may establish synergy between the phages in the cocktail, where one enhances the characteristics of another, resulting in improved phage adsorption and robustness in pathogen lysis rates. Unlike the antibiotics and copper-based disease management approaches, which pose serious threats to the environment and human beings as well, a phage cocktail therapy is an eco-friendly approach that provides new insights to control the infections of widespread pathogens, including *P. syringae* pv. *porri* [76], *X. campestris* pv. *campestris* [77], *X. euvesicatoria* [78], *Pectobacterium carotovorum* subsp. *carotovorum* [79]. More recent developments in phage cocktail therapy against plant pathogenic bacteria have been included in Table 1. Moreover, a moko disease of banana, plantain, and heliconia plants was reported as an epidemic in Brazil and Latin America due to the diversity of the disease-causing pathogen *R. solanacearum* and indeterminate disease management strategies [200]. Nowadays, a cocktail of two lytic phages, such as M5 and M8, is formulated and dynamically applied in Colombia and found to be effective against all resistant strains of *R. solanacearum* [201]. Similarly, a phage cocktail (NJ-P3, NB-P21, NC-P34, and NN-P42) effectively killed the majority of *R. solanacearum* in the soil, rendering phage resistance enhancement and reducing the growth and competitiveness of phage-resistant bacterial pathogens in the rhizosphere. There is a higher rise in microbiota diversification and an enrichment of bacterial species that are antagonistic to *R. solanacearum*, both of which lead to an extremely low pathogen density [80]. In carrot fields, an antimicrobial resistant pathogen, including the *P. aeruginosa* (PAO1) encoding cm1A, a chloramphenicol resistant gene, is effectively controlled by the application of a cocktail of four polyvalent phages, such as ϕYSZ1, ϕYSZ2, ϕYSZ3, and ϕYSZ4 [202].

Phage cocktail therapy in conjunction with other antimicrobial agents, including antibiotics and plant SAR inducers, which help to reduce disease severity, has revolutionized sustainable disease management and received a lot of attention, showing great potential [177,179,181]. Applications of the phage cocktail with acibenzolar-S-methyl (ASM) efficiently reduces the disease incidence of bacterial spot of tomato caused by *X. campestris* pv. *vesicatoria* and bacterial leaf blight of onion induced by *X. axonopodis* pv. *allii* under greenhouse as well as field conditions [52]. Phage-encoded peptidoglycan hydrolases (PGHs), called endolysins and holins, which trigger enzymatically degradation of the host bacterium’s PG, have brought voluminous advances in phage cocktail therapy of wide-ranging antimicrobial-resistant pathogens. Based on catalytic activity, these endolysins have been categorized into five groups: endopeptidases, transglycosylases, N-acetylmuramidases, N-acetylmuramoyl-L-alanine amidases, and endo-β-N-acetylglucosaminidases [184,208,209,210,211,212,213,214]. These endolysins demonstrate great bacteriolytic range not only within a species but also across the other genera. Indeed, a phage Xop411 of *X. oryzae* encodes gp21 protein that kills *Xanthomonas* species as well as other aggressive bacterial pathogens, including *Stenotrophomonas maltophilla* and *P. aeruginosa* [215]. The application of phage CMP1 on tomato plants may induce the expression of the endolysins gene (*lys*) that triggers resistance in plants against *C. michiganensis* and reduces diseases severity as well as the number of bacterial cells in the xylem sap and leaf extracts [216]. On the other hand, several pathogenic bacterial communities use extracellular matrix, including EPS, to form biofilms on the surface of plants, which has notable implications in plant disease control strategies [217,218], because many phages have limited access to bacteria inside these structures. However, phage-encoded enzymes, such as endolysins, depolymerases, holins, and other PGHs, may offer a concrete solution to biofilm degradation and eradication. This is a dynamic approach for phage cocktail-based biocontrol against resistant phytopathogenic bacteria [219,220,221,222]. The bactericidal potential of some endolysins is not reported against gram-negative bacteria because their outer membrane prevents the PG from hydrolysis by phage endolysins. Therefore, several approaches have been developed to circumvent this disadvantage, such as a cocktail of two or more phages encoding endolysins that meet each other’s limitations to produce effective bactericidal actions against a variety of bacterial pathogens [190]. Thus, these strategies may open new frontiers for developing drugs, vaccines, and designing phage cocktails for large numbers of antimicrobial-resistant phytobacteria.

## 7. The Issue of Host Resistance and Advantages of Phage-Mediated Biocontrol Strategies

**Figure 4:**
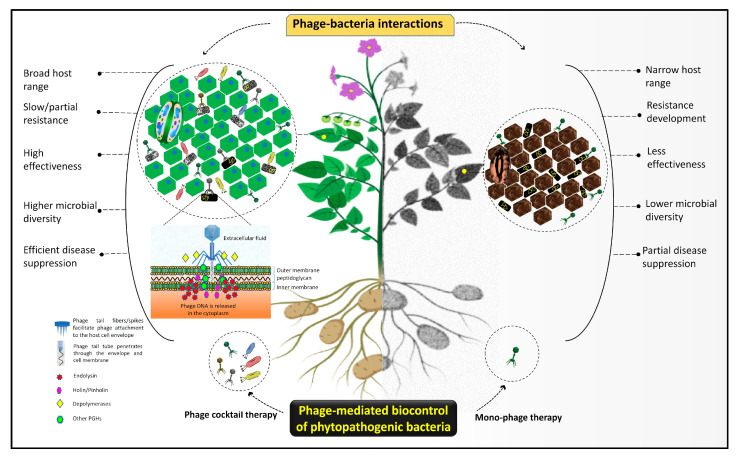
A schematic model comparing the mechanism and efficiency of mono-phage and phage cocktail therapies for the management of plant pathogenic bacteria (created with BioRender.com, accessed on 2 December 2021).

Plant pathogenic bacteria undergo enormous evolutionary pressure from phages. To cope with this pressure, bacteria have evolved with diversified immune mechanisms, including innate and adaptive, which evade the phage infection through a variety of phage resistance actions (Figure 3) [96,142,196,223,224,225,226,227,228]. For the identification and characterization of the anti-phage underlying mechanisms, newly discovered CRISPR-Cas and RM systems have been established. A comprehensive review has been published on anti-phage mechanisms and counter-defense strategies of phages [229]. The alteration or loss of the bacterial cell surface and bacterial extracellular matrix-mediated blockage of receptors may also result in phage resistance, leading to the inhibition of phage penetration, the production of modified restriction endonucleases degrading phage DNA, or the inhibition of phage intracellular development [96,99]. For example, to avoid the adsorption of particular phages SA039 and PP01 of *Staphylococcus aureus* (SA003) and *E. coli* (O157:H7), eliminate the OmpC protein and β-GlcNAc residue on the WTA, respectively. As a counter-adaptation, phages evolve and adopt point mutation in receptor binding proteins (RBPs) to adapt receptors, enabling them to target resistant hosts. For example, a mutation in *gp38*, encoding a tail protein in coliphage (PP01), empowers the phage to infect OmpC deficient receptors [230,231]. Similarly, a mutation in *orf103*, encoding RBP, enables staphylococcal phage (ϕSA012) to target phage-resistant *S. aureus* (SA003) [232]. Superinfection exclusions are regulated by the *ltp* gene in temperate phages e.g., *Streptococcus thermophiles*, encoding the membrane lipoprotein Ltp that interacts with the channel-forming protein to inhibit the entry of phage DNA into the host cytoplasm [233]. Unlike CRISPR-Cas, DISARM, BREX, and RM systems, which target any phage DNA regardless of the origin and inhibit its entry and replication into a phage resistant bacterium, abortive infection encoded by mobile genetic elements (plasmids and temperate phages) is phage specific and targets the phage infection cycle at different stages. A detailed review on the phage–host arms race has been published [196]. To combat these defensive mechanisms, phages have also deployed a multifaceted array of counter-defense approaches. Phages encode extraordinary proteins, including RBPs, PGHs, endolysins, and depolymerases, which enable the phage-mediated biocontrol strategy against wide-ranging antimicrobial-resistant pathogens [190]. For example, the application of phages, such as the CMP1 encoding lys and Xop411 encoding gp21, can kill *Clavibacter* and *Xanthomonas* species in plants [215,216]. A resistant pathogen, *X. fastidiosa*, causing disease epidemics was effectively controlled by the combination of four lytic phages [62,69,70]. This can lead to the development of phage cocktails in which individual members work in synergy to eradicate the target resistant bacterial pathogen. Moreover, phage cocktails in association with antibiotics and antimicrobial agents have great potential to eradicate resistant phytobacteria from phytosystem and enhance agricultural production [52]. Therefore, the interaction between phages and resistant phytobacteria signifies the potential impact of phage cocktails in biocontrol-mediated disease management and enables the development of multidimensional cocktails based on phage invaders that evade the resistant mechanisms of these plant pathogenic bacteria. A schematic model of phage–phytobacteria interactions demonstrates the comparative efficiency of single vs. multiple phages (cocktail) as biocontrol agents of bacterial plant diseases (Figure 4).

## 8. Knowledge Gaps and Future Directions

Plant phage cocktails have been explored as promising biocontrol therapeutics for the management of overwhelming bacterial diseases in plants without intimidating fauna and flora, and for the investigation of evolutionary interactions between phage and resistant phytobacteria, as highlighted in this communication. There are diversified mechanisms of phage resistance in bacterial pathogens and emerging strategies that phages employ to evade these systems for successful infections [59,196,227,229]. This knowledge is facilitating synthetic biology in the development of multidimensional phage-mediated therapeutics and novel biotechnological platforms. Regardless of the significant progress, there is still a long way to go in exploring pathogenic bacterial defenses and phage counter-adaptations. Furthermore, the recent discoveries reveal that our understanding of the defense arsenal is insufficient, mandating additional systematized methodologies for their evaluation. To advance the phage cocktail therapy, both sides of the arms race, the bacteria and the phages, must be taken into consideration. In the case of plants, critical gaps exist in molecular characteristics, formulations, and applications of phage cocktail therapeutics against wide-ranging resistant bacterial pathogens. However, fundamental questions remain: (i) From the application perspective, how do phages diffuse systemically in the plant to target specific bacterial pathogens? (ii) How can phages overcome their self-interaction in a cocktail to achieve synergistic interaction against broad-spectrum bacterial control? (iii) What are the consequences and repercussions for prophage–host interaction dynamics and ecosystem function? (iv) How does lysogen abundance change across space, time, and taxa? (v) The genomes of phages are still relatively underexploited. Which phage-encoded novel proteins with therapeutic potential will be discovered in the future, and what applications and functions will they deliver? (vi) Which innovative and exceptional biotechnological tools will be developed using existing and newly reported phage-encoded proteins, and what influence will they have on the formulation of phage cocktail therapeutics? The knowledge of phage–bacterium interactions in natural environments is still debatable, and further research is required to understand how biodiversity and abiotic factors influence phage–bacterium ecological and evolutionary dynamics. Furthermore, it is becoming obvious that phage and bacterial communities may have a significant influence on their eukaryotic hosts [234,235].

Nowadays, only lytic phages are used in phage cocktail therapy for the management of plant bacterial diseases, but there is a question mark over the potential impact of temperate and filamentous phages. Their replication cycle makes them unsuitable as biocontrol agents for plant disease management, although they can be engineered and manipulated to become virulent or used as a vector for genetic elements for virulence factor disruptions or antimicrobial susceptibility restoration [47,59,169,177,183,190]. Moreover, in phage-mediated pathogen detection, engineered phages are used to insert marker genes into the genomes of targeted bacteria. Therefore, irrespective of reporter phages, whether lytic or lysogenic, potentially, they may still detect the target bacterial pathogen [236]. Recently, our understanding of phages has been advancing with computational genetic programs and availability of increased sequencing data, enabling the success of bioinformatics platforms to establish more systemic approaches, which may facilitate phage cocktail therapy against resistant phytobacteria [237,238,239,240,241]. We can concentrate on the genes that are likely to disrupt bacterial immunity. For example, prophage-encoded genes regulating phage defense are located in certain genomic regions, and comparative genomics of phage families has facilitated their discovery. Additionally, early expressed genes are frequently involved in anti-defense or bacterial takeover [190]. In order to understand therapeutics, ecological significance, and the biotechnological repercussions of phages, mechanistic investigations must be complemented with high-throughput experimentations to elucidate how molecular events scale to global microbial processes. It is plausible that new information on the function of phage-mediated biocontrol therapy will emerge, and those gigantic discoveries will come shortly, some of which will be powerful enough to revolutionize medicinal, agricultural, and industrial biotechnologies.

## 9. Conclusions

Phage cocktail therapy has heralded a revolutionary track in the management of various plant bacterial diseases, resulting in increased agricultural productions to sustain the food supply chain. Biotechnological platforms have provided new insights into the development of multifaceted phage cocktails, capable of targeting resistant plant pathogenic bacteria with extremely high efficiency. Several commercial phage-based biocontrol therapeutics have reached the market to mitigate devastating bacterial diseases, such as Agri-phage to control fire blight of pear and apple trees and bacterial spot of tomatoes and peppers, Biolysis to eradicate soft rot disease of potato tubers, and Erwiphage for the management of fire blight of apple trees [12]. In addition, the ectopic expression of phage-encoded proteins frequently enhances the plant’s resistance to bacterial pathogens [216]. Although agrochemicals, including antibiotics and copper-based microbial compounds, are still applied in the field to combat bacterial plant diseases, phage cocktail application has the potential to reduce the number of agrochemicals employed or to replace these agrochemicals for the management of bacterial plant diseases. Therefore, more phage cocktails for several bacterial pathogens need to be assembled on the basis of field experiments rather than controlled conditions.

## Figures and Tables

**Table 1:** Phage cocktails, which have recently been applied to effectively control plant-pathogenic bacterial diseases.

Bacterial Pathogen	Disease	Host	Phage Cocktails	Treatment Effects	Year	Reference
*Ralstonia* *solanacearum*	Bacterial wilt	Tomato	vRsoP-WM2, vRsoP-WF2, and vRsoP-WR2	The development of symptoms is significantly inhibited.	2019	[47]
*Erwinia amylovora*	Fire blight	Apple and pear	ϕEa1337-26, Eh21-5 and ϕEa2345-6	Reduces disease infection predominantly.	2011	[159]
*Xylella fastidiosa* subsp. *fastidiosa*	Pierce disease	Grapevine	Polyvalent phages	In laboratory investigations, therapeutic and prophylactic phage administration greatly reduces disease symptoms.	2015	[62]
*Dickeya solani*, *Pectobacterium* *Atrosepticum*, *Pectobacterium* *carotovorum* subsp. *Carotovorum*	Soft rot	Potato	ϕPD10.3 and ϕPD23.1	The soft rot disease is decreased by 80–95% when the phage cocktail and pathogens have been co-inoculated on potato tuber slices and entire tubers.	2015	[82]
*Pectobacterium* *atrosepticum* *Pectobacterium* *carotavorum* subsp. *Carotavorum*	Soft rot and blackleg	Potato	fMA1, fMA1A, fMA2, fMA5, fMA6 and fMA7	Soft rot disease development is considerably reduced when phages are applied to the soil. Tissues maceration is also inhibited significantly.	2020	[79]
*Pectobacterium* *atrosepticum*	Soft rot	Potato	Phage Nepra, Lelidair, Nobby, Slant, Gaspode And Momine	Under field condition, disease severity and incidence are reduced by 64.2% and 61.3%, respectively.	2019	[203]
*Pectobacterium* *atrosepticum*	Soft rot	Potato	vB_PatP_CB1, vB_PatP_CB3, and vB_PatP_CB4	The percentage of decaying tissue is reduced significantly.	2018	[204]
*Pseudomonas* *syringae* pv. *actinidiae*	Bacterial canker	Kiwi	CHF1, CHF7, CHF19, and CHF21	In a greenhouse, phage cocktail treatment resulted in a 75% reduction of bacterial titer in leaves 24 h after inoculation.	2020	[205]
*Pseudomonas* *syringae* pv. *syringae, P.* *syringae* pv. *morsprunorum* *race 1*, and *race* *2*	Bacterial canker	Cherry trees	MR1, MR2, MR4, MR5, MR6, MR7, MR8, MR12, MR13, MR14, MR15, MR16, and MR18	In a field, 15–40% reduction in bacterial titers has been reported in bean leaves and in cherry twigs and seedlings as well.	2020	[206]
*Pseudomonas* *syringae* pv. *porri*	Bacterial blight	Leek	KIL3b and KIL5	Reduces bacterial concentration 100-fold significantly. In LPS, bacterial resistance mutations have a cost in terms of viability.	2020	[207]
*Ralstonia* *solanacearum*	Bacterial wilt	Potato	P-PSG-1, P-PSG-2, P-PSG-3, P-PSG-7, P-PSG-8, and P-PSG-9	In preventative therapy, wilt is reduced by 80%. In curative therapy, there is a delay in disease development. In a soil assay, phage spraying resulted in a 98% reduction in bacterial titer after one week.	2017	[75]
*Ralstonia* *solanacearum*	Bacterial wilt	Potato	vRsoP-WF2, vRsoP-WM2, and vRsoP-WR2	Symptom development has been greatly decreased in both the green house and the field.	2019	[47]
*Xanthomonas citri* subsp. *citri*	Citrus canker	Grapefruit	ϕXV3-21, ϕXaacF1, and ccϕ19-1	ϕXaac F1 with ϕXV3-21 and ccϕ19-1 phage is reported to reduce disease symptoms by 58% in first and 69% in second phase of application. The ϕXaac F1 is most persistent in the phyllosphere and multiplies more efficiently than the other two phages.	2018	[141]

## Data Availability

Not applicable.
